# Poor Man's Crown

**Published:** 1900-01

**Authors:** J. N. Davenport

**Affiliations:** Northampton, Mass.


### ARTICLE VII.

### POOR MAN'S CROWN.

DR. J. N. DAVENPORT, NORTHAMPTION, MASS.

The materials of which the finished crown is composed are an ordinary rubber, plain tooth, German silver wire hammered into shape that the opening in the root requires, and the ordinary vulcanizing rubber of any color decided upon. The *modus operandi* is very simple. The root-canal being first prepared, a plain, rubber tooth is selected and ground to the gum. The end of the root is ground fairly smooth, but one peculiarity about the method is that, if the palatine or lingual portion of the root stands rather high and is fairly strong, it is not necessary to cut it to the gum, as for most other methods it being possible to make the crown conform to any irregularity of the root. A piece of German silver wire, of suitable size, is hammered on the anvil to the proper shape and fitted to the root-canal. The hammering gives additional stiffness, and makes the wire so rigid that there is no possibility of bending it with the oridinary force which comes upon a crown. One end of the German silver is then made jagged with the file and passed between the pins of the tooth, which are bent to hold it. Red, pink or white vulcanizing rubber in small pieces is now packed upon the pins and over the wire with a warm instrument, and in sufficient quantity to form a shoulder which will cover the end of the root. This unfinished affair is then put into place, the pin going to its full extent into the root-canal, and the porcelain pressed to its proper position. Before it is put into place it is held over the spirit-lamp to soften the surface of the rubber, and the moistened thumb and finger will form the rubber by pressure accurately to the end of the root. It can be taken, the surplus of soft rubber trimmed off, and put back again, half a dozen times, if necessary, within a few minutes, until everything seems right, when it is finally removed, invested in plaster in the flask, vulcanized and finished. There are many ideas which can be applied to this method. It has been suggested by Dr. Davenport that wax instead of rubber could be used for the first fitting, particularly when two or three root-canals were to be used, and after everything was adjusted the waxed crown could be placed in the flask, the flask opened, the wax taken out, and rubber packed in. Then, too, bicuspids can easily be formed and made quite sightly by the use of white rubber for the inner cusp. These crowns should be secured to place with zinc phosphate.—*International.*

